# EVALUATING THE EVIDENCE BASE OF A STAFFING GUIDELINE FOR DUTCH NURSING HOMES

**DOI:** 10.1093/geroni/igz038.1399

**Published:** 2019-11-08

**Authors:** Ramona Backhaus, Hilde Verbeek, Bram De Boer, Erik Van Rossum, Jos Schols, Jan Hamers

**Affiliations:** 1 Maastricht University, Maastricht, Netherlands; 2 Maastricht University, Maastricht, Limburg, Netherlands; 4 Zuyd Hogeschool, Heerlen, Limburg, Netherlands

## Abstract

Related to the Dutch nursing home quality framework implemented in 2017, a staffing guideline was developed, aimed at assisting nursing homes to adequately staff their wards. For the Dutch Ministry of Health, we investigated the evidence base of this guideline. We critically reviewed scientific literature (n=65) and interviewed (inter)national experts (n=8) and potential guideline users (n=5). We found that departing a quality improvement dialogue directly from teams, clients and their families is positive. However, weaknesses were identified as well. Several risks exist for employees to adequately assess resident needs. Furthermore, buy-in is needed from board level to develop a vision on which competencies and amount of staff are needed to fulfill these needs. Examples of guideline improvement recommendations were assisting teams in how to assess resident needs, critically reflect on care provision and considering a role for (top)management. Overall, it was concluded that the value of the guideline was limited.

